# Nicotine Promotes Proliferation of Human Nasopharyngeal Carcinoma Cells by Regulating α7AChR, ERK, HIF-1α and VEGF/PEDF Signaling

**DOI:** 10.1371/journal.pone.0043898

**Published:** 2012-08-31

**Authors:** Dingbo Shi, Wei Guo, Wangbin Chen, Lingyi Fu, Jingshu Wang, Yung Tian, Xiangsheng Xiao, Tiebang Kang, Wenlin Huang, Wuguo Deng

**Affiliations:** 1 State Key Laboratory of Oncology in South China, Sun Yat-Sen University Cancer Center, Guangzhou, China; 2 State Key Laboratory of Targeted Therapy Drug of Guangdong, Guangzhou, China; 3 Institute of Cancer Stem Cell, Dalian Medical University Cancer Center, Dalian, China; University of Hawaii Cancer Center, United States of America

## Abstract

Nicotine, the major component in cigarette smoke, can promote tumor growth and angiogenesis, but the precise mechanisms involved remain largely unknown. Here, we investigated the mechanism of action of nicotine in human nasopharyngeal carcinoma (NPC) cells. Nicotine significantly promoted cell proliferation in a dose and time-dependent manner in human NPC cells. The mechanism studies showed that the observed stimulation of proliferation was accompanied by the nicotine-mediated simultaneous modulation of α7AChR, HIF-1α, ERK and VEGF/PEDF signaling. Treatment of NPC cells with nicotine markedly upregulated the expression of α7AChR and HIF-1α proteins. Transfection with a α7AChR or HIF-1α-specific siRNA or a α7AChR-selective inhibitor significantly attenuated the nicotine-mediated promotion of NPC cell proliferation. Nicotine also promoted the phosphorylation of ERK1/2 but not JNK and p38 proteins, thereby induced the activation of ERK/MAPK signaling pathway. Pretreatment with an ERK-selective inhibitor effectively reduced the nicotine-induced proliferation of NPC cells. Moreover, nicotine upregulated the expression of VEGF but suppressed the expression of PEDF at mRNA and protein levels, leading to a significant increase of the ratio of VEGF/PEDF in NPC cells. Pretreatment with a α7AChR or ERK-selective inhibitor or transfection with a HIF-1α-specific siRNA in NPC cells significantly inhibited the nicotine-induced HIF-1α expression and VEGF/PEDF ratio. These results therefore indicate that nicotine promotes proliferation of human NPC cells *in vitro* through simultaneous modulation of α7AChR, HIF-1α, ERK and VEGF/PEDF signaling and suggest that the related molecules such as HIF-1α might be the potential therapeutic targets for tobacco-associated diseases such as nasopharyngeal carcinomas.

## Introduction

Nasopharyngeal carcinoma (NPC) has the highest occurrence in Southeast Asia and is one of the leading causes for cancer mortality in Cantonese region of Southern China [1], [2]. It is well known that tobacco use is one of the most important risk factors for the development of cancer. Nicotine, a major component of cigarette smoke, has been shown to be involved in the initiation, promotion, and even progression of several tumors including lung cancer, gastric cancer, pancreatic cancer, and head and neck cancers [3]–[9]. However, the effect of nicotine on tumorigenesis and angiogenesis of human NPC and the mechanism of action of nicotine involved remain largely unknown.

Several lines of evidence suggest that nicotine exerts its cellular functions through nicotinic acetyl-choline receptors (nAChRs), which are widespread in neurons, neuromuscular junctions and many tumor cells [10], [11]. Especially, previous studies have shown that nicotine functions through its interaction with α7AChR [12], [13]. α7AChR is a kind of integral membrane protein, which is highly expressed in a portion of tumors and closely associated with cancer cells growth, migration, angiogenesis, and apoptosis [14]. However, no information has been available about whether nicotine also affects proliferation of human NPC cells through regulation of the α7AChR.

Hypoxia-inducible factor-1 (HIF-1) is a transcription factor which activates the expression of a number of genes involved in diverse aspects of cellular and physiologic processes [15], [16]. It includes two forms, HIF-1α and HIF-1ß. The function of HIF-1α is tightly regulated by cellular oxygen concentration. Under hypoxic conditions, HIF-1α forms a heterodimer with HIF-1ß, and binds to the hypoxia-responsive elements of the promoters to activate downstream hypoxia-responsive genes, including vascular endothelial growth factor (VEGF), to increase angiogenesis and tumor metastasis or to promote cancer cell proliferation and migration [17]. By binding to the hypoxia-responsive elements on VEGF promoter, HIF-1 leads to the transcriptional activation of the VEGF gene [18], [19]. The potent angiogenic inhibitor pigment epithelium-derived factor (PEDF) counterbalances the effect of VEGF [20]. The activity of HIF-1α is up-regulated by a variety of nonhypoxic signals, including the activation by several oncogenic pathways such as Src, HER-2, Ha-Ras, and mitogen-activated protein kinase (MAPK) signaling pathways [21]. HIF-1α is overexpressed in many human cancers including NPC, and several lines of evidence indicated its essential role in tumorigenesis [22], [23]. HIF-1α has also been shown to be activated by phosphatidylinositol3-kinase (PI3K) pathway in HER-2 overexpressing cells [24]. However, the detailed mechanisms of action of HIF-1α in NPC tumorigenesis and the nicotine-mediated regulation of HIF-1α, MAPK and VEGF/PEDF signaling in human NPC cells is still largely unknown.

In this study, we evaluated the effect of nicotine on cell proliferation in various NPC cells. The underlying mechanisms of nicotine promoting NPC cell proliferation were also investigated. We showed that nicotine significantly promoted cell proliferation by simultaneously regulating the α7AChR, HIF-1α, ERK, and VEGF/PEDF signaling in the tested human NPC cell lines. Our findings provide new insights into understanding the precise mechanisms of action of nicotine and exploring the potential therapeutic targets for tobacco-associated diseases such as nasopharyngeal carcinomas.

## Results

### Nicotine Promotes NPC Cell Proliferation in a Dose and Time-dependent Manner

**Figure 1:**
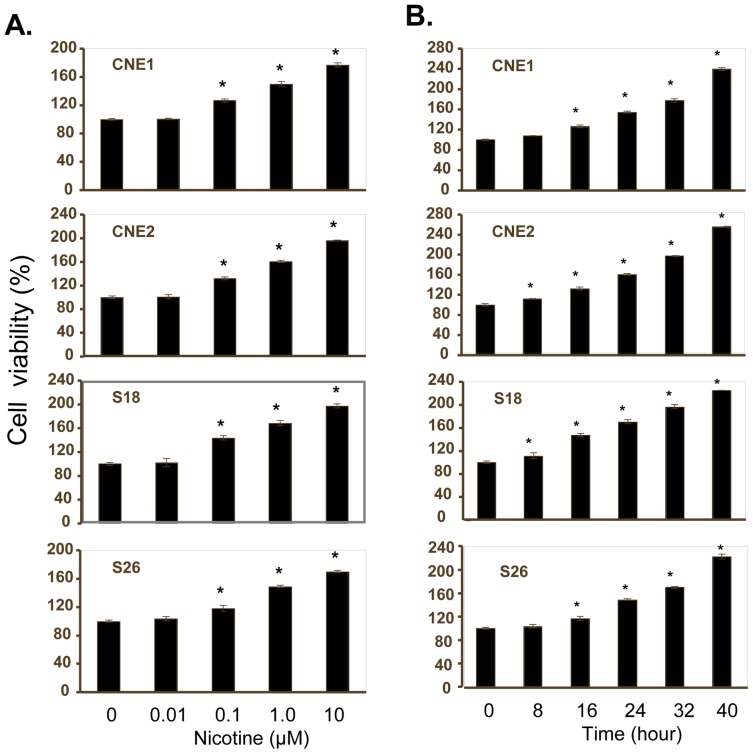
Nicotine promotes proliferation of NPC cells. Human CNE1, CNE2, S18 and S26 cells were treated with nicotine at the doses of 0.01–10 µM for 72 hours **(A)** or at the concentration of 10 µM for 8–40 hours **(B)**. The cell viability was determined by MTT assay **(A)**. Cells treated with vehicle control DMSO were used as the referent group with cell viability set at 100%. The percent cell viability in each treatment group was calculated relative to cells treated with vehicle control. The data are presented as mean ± SD of three separate experiments. *, *P*<0.05, significant differences between treatment groups and control groups.

To determine whether nicotine may regulate the proliferation of NPC cells, we examined the effect of nicotine on cell viability in various kinds of human NPC cell lines, including nasopharyngeal high differentiated squamous epithelium carcinoma cell line CNE1, nasopharyngeal low differentiated squamous epithelium carcinomas cell line CNE2 as well as its clones S18 and S26, by a MTT analysis. The results showed that treatment with nicotine at the concentrations of 0.1–10 µM for 72 hours significantly promoted cell proliferation in a concentration-dependent manner, resulting in a 20–100% increase in cell viability in all four kinds of NPC cell lines (Fig.1A). We also examined the effect of nicotine on NPC cell proliferation at the concentration of 10 µM at a serial of different treatment time (8–40 hours), and found that nicotine significantly promoted cell proliferation in a time-dependent manner in all four kinds of NPC cell lines (Fig.1B).

### Nicotine Upregulates α7AchR Expression

**Figure 2:**
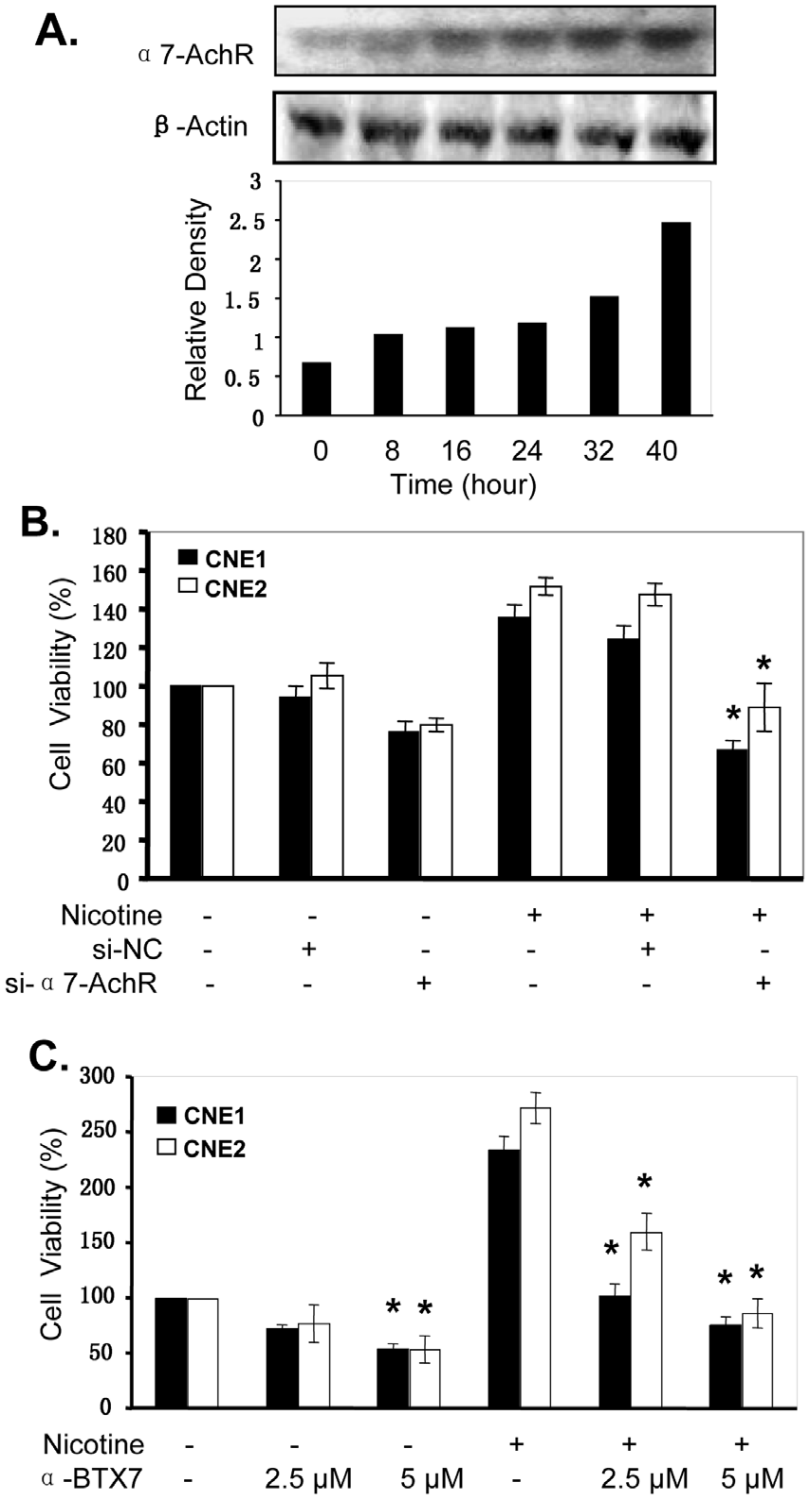
Nicotine upregulates α7AChR expression in NPC cells. **(A),** Human CNE1 cells were treated with nicotine at the concentration of 10 µM for 8–40 hours. The expression of α7AChR protein levels were detected by Western blotting. β-Actin was used as a control for sample loading. The bottom panels are the relative densities of the α7AChR protein bands to β-Actin. **(B, C)**, Human NPC cell lines CNE1 and CNE2 were transfected with the α7AChR-specific siRNA (si-α7AChR, 100 nM) **(B)** or the α7AChR-selective inhibitor α-BTX (2.5 and 5 µM) **(C)** for 4 hours, and then treated with nicotine at 10 µM. At 72 hours after treatment, cell viability was determined by MTT analysis. The scrambled non-specific control siRNA (si-NS) was used as a negative control. The percent cell viability in each treatment group was calculated relative to cells treated with the vehicle control. The data are presented as the mean ± SD of three separate experiments. *, *P*<0.05, significant differences between treatment groups and control groups.

α7AChR, a kind of nicotinic acetyl-choline receptors, is closely associated with cancer cell growth and angiogenesis [25], [26]. To determine whether the nicotine-mediated promotion of cell proliferation in NPC cells is also mediated through α7AchR signaling, we analyzed the effect of nicotine on the expression of α7AchR protein in CNE1 cells by Western blot. As shown in Fig. 2A, treatment of nicotine at the dose of 10 µM for 8–40 hours promoted the expression of α7AchR protein in a time-dependent manner.

To further confirm the involvement of the α7AchR signaling pathway in the nicotine-mediated promotion of NPC cell proliferation, we blocked α7AchR protein expression by transfecting CNE1 and CNE2 cells with a α7AchR-specific siRNA (si-α7AchR) and evaluated its effects on nicotine-mediated promotion of cell proliferation. By comparison with the scrambled non-specific control siRNA (si-NS), transfection with si-α7AchR (100 nM) considerably inhibited cell proliferation (Fig. 2B). Moreover, si-α7AChR transfection significantly reduced the nicotine-mediated promotion of cell proliferation in both CNE1 and CNE2 cells (Fig. 2B).

We also analyzed the effect of α-BTX, a α7AChR-selective inhibitor, on nicotine-mediated promotion of cell proliferation in NPC cells. Treatment of CNE1 and CNE2 cells with nicotine increased cell viability, but pretreatment with α-BTX (2.5 and 5 µM) significantly attenuated nicotine-mediated promotion of cell proliferation (Fig. 2C). These results indicate that nicotine-induced NPC cell proliferation might be partially through regulation of the α7nAchR signaling.

### Nicotine Promotes HIF-1α Expression

**Figure 3:**
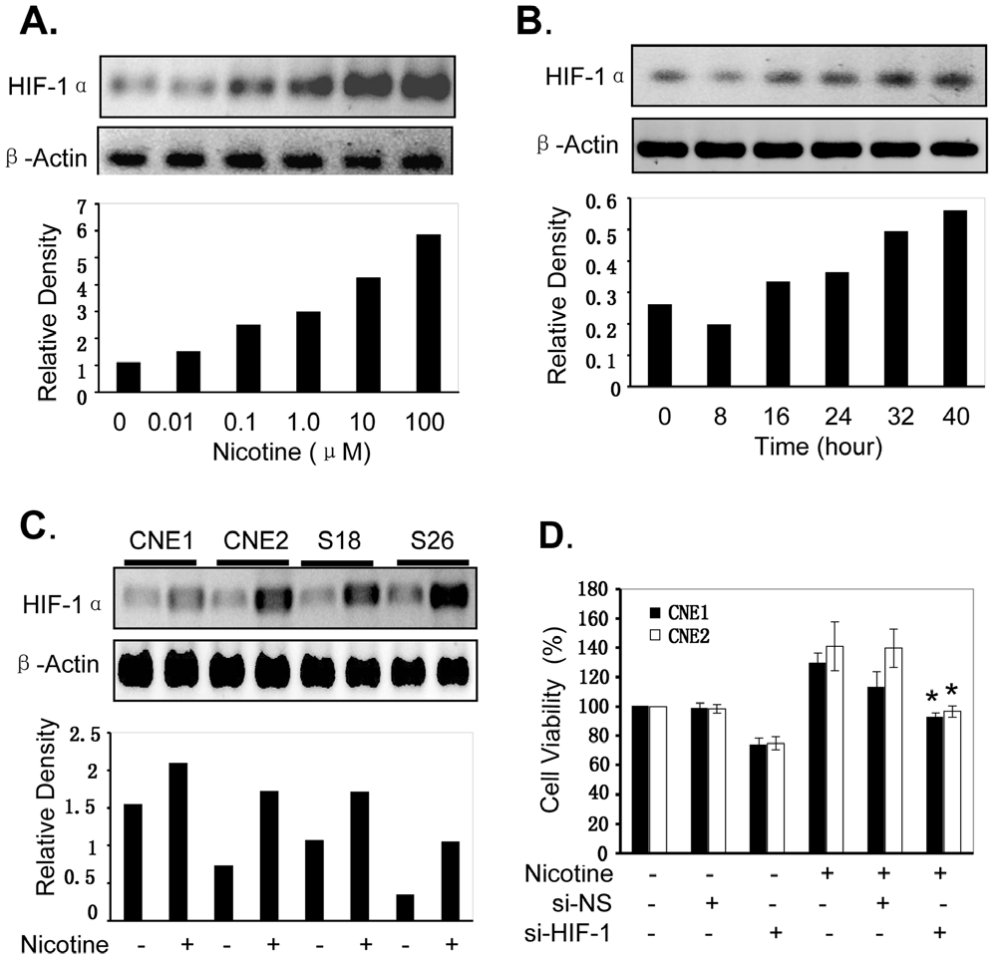
Nicotine activates HIF-1α signaling in NPC cells. Human CNE1 cells were treated with nicotine at the doses of 0.01–100 µM for 24 hours **(A)** or at the concentration of 10 µM for 8–40 hours **(B)**, or human CNE1, CNE2, S18 or S26 cells were treated with nicotine at the doses of 10 µM for 24 hours **(C)**. The expression of HIF-1α protein levels were detected by Western blotting. β-Actin was used as a control for sample loading. The bottom panels are the relative densities of HIF-1α protein bands to β-Actin. **(D)**, Human CNE1 and CNE2 cells were transfected with the HIF-1α-specific siRNA (si-HIF-1α, 100 nM) for 4 hours, and then treated with nicotine at 10 µM. At 72 hours after treatment, cell viability was determined by MTT analysis. The scrambled non-specific control siRNA (si-NS) was used as a negative control. The percent cell viability in each treatment group was calculated relative to cells treated with the vehicle control. The data are presented as the mean ± SD of three separate experiments. *, *P*<0.05, significant differences between treatment groups and control groups.

HIF-1α overexpression is associated with angiogenesis and tumor cell proliferation and invasion [27]. We determined whether the effect of nicotine in promoting NPC cells proliferation was realized through activating HIF-1α pathway. The effect of nicotine on HIF-1α protein expression was analyzed using western blot. As shown in Fig. 3A and 3B, treatment with nicotine significantly promoted the expression of HIF-1α protein in a concentration and time-dependent manner in CNE1 cells. Nicotine also significantly upregulated HIF-1α protein expression in other kinds of NPC cell lines such as CNE2 and its colons S18 and S26 (Fig. 3C).

To confirm the role of nicotine in regulating HIF-1α signaling in NPC cells, we blocked HIF-1α expression by transfecting cells with a HIF-1α-specific siRNA (si-HIF-1α) and evaluated its effects on nicotine-mediated promotion of cell proliferation in CNE1 and CNE2 cells. By comparison with the scrambled non-specific control siRNA (si-NS), transfection with si-HIF-1α at the dose of 100 nM significantly inhibited cell proliferation induced by nicotine (Fig. 3D), indicating that the nicotine-mediated promotion of NPC cells growth might also partially be regulated by activating HIF-1α signaling pathway.

### Nicotine Induces ERK/MAPK Activation

**Figure 4:**
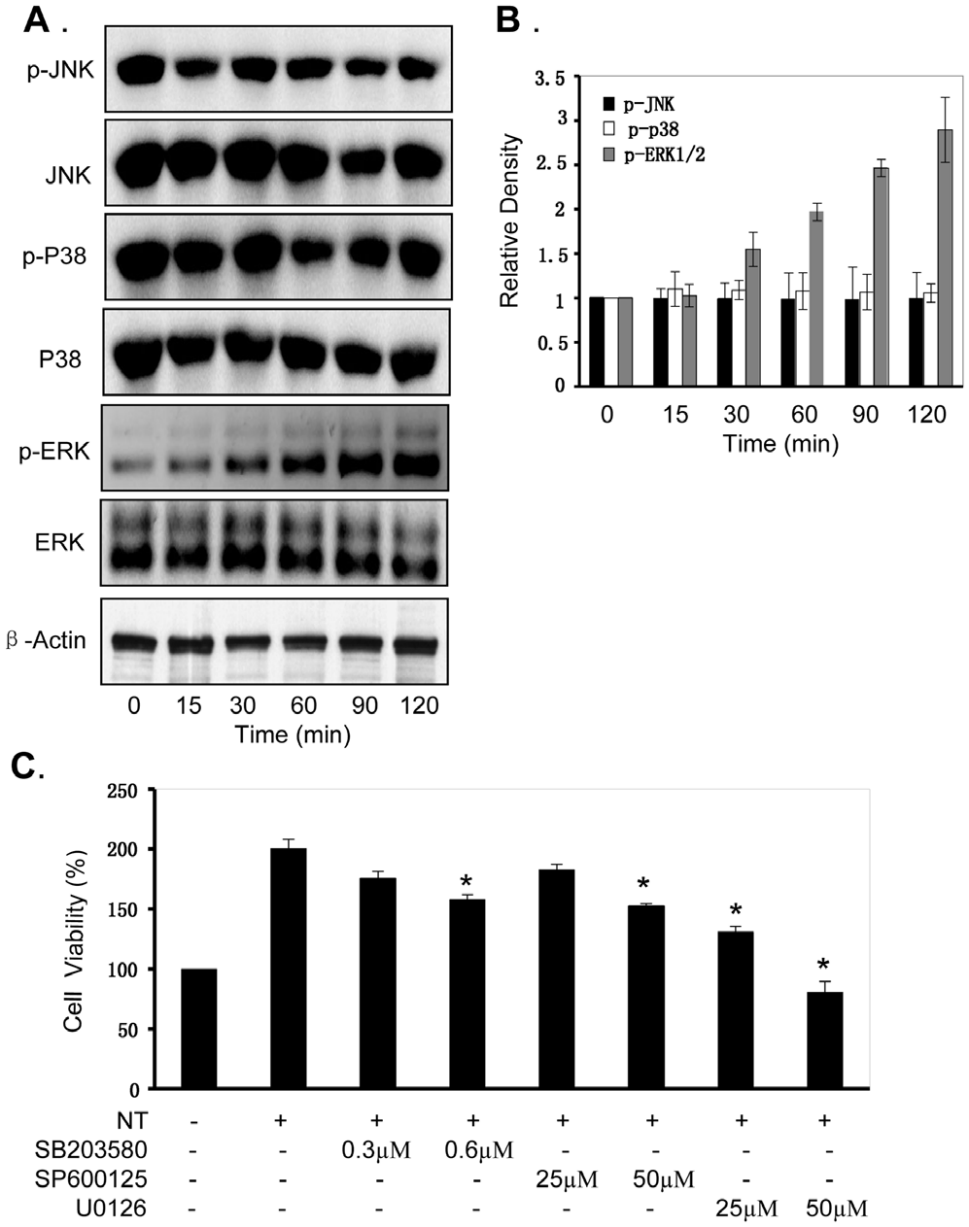
Nicotine induced ERA/MAPK activation in NPC cells. **(A, B),** Human CNE1 cells were treated with nicotine at the concentration of 10 µM for 15–120 minutes. The expression of the phosphorylated or total protein of ERK1/2, JNK and P38 **(A)** were detected by Western blotting. β-Actin was used as a control for sample loading. The relative densities of ERK1/2, JNK and p38 proteins to β-Actin **(B)** were analyzed. **(C),** Human CNE1 cells were treated with the ERK-selective inhibitor U0126 (25 and 50 µM), JNK inhibitor SB203580 (300 and 600 nM) or p38 inhibitor SP600125 (25 and 50 nM), respectively, for 4 hours, and then treated with nicotine at 10 µM. At 72 hours after treatment, cell viability **(C)** was determined by MTT analysis. The percent cell viability in each treatment group was calculated relative to cells treated with the vehicle control. The data are presented as the mean ± SD of three separate experiments. *, *P*<0.05, significant differences between treatment groups and control groups.

It has shown that MAPK is required for the transactivation activity of HIF-1α [21]. To determine whether nicotine-mediated promotion of NPC cell proliferation is through the activation of MAPK signaling pathway, we evaluated the effect of nicotine on the activation of ERK, JNK and P38, three key signaling proteins in MAPK pathway, by western blot. As shown in Fig. 4A, treatment with nicotine at 10 µM for 15–120 minutes significantly increased the levels of the phosphorylated ERK1/2 protein in a time-dependent manner, whereas the levels of total ERK1/2 protein did not change. By contrast, nicotine did not alter the levels of JNK, P38 and their phosphorylated forms.

To further confirm the nicotine-induced activation of the ERK/MAPK pathway in NPC cells, we next analyzed the effects of the ERK, JNK and P38-selective inhibitors (U0126, SB203580, SP600125) on nicotine-mediated proliferation promotion in NPC cells. Pretreatment with JNK or P38 inhibitor (SB203580 or SP600125) slightly reduced the nicotine-mediated promotion of cell proliferation (Fig. 4C). By contrast, pretreatment with an ERK inhibitor (U0126) dramatically inhibited the effect of nicotine on promotion of cell proliferation (Fig. 4C). These results indicate that ERK signaling is an important target of nicotine and the cell proliferation promotion by nicotine in NPC cells might be also partially mediated by activating the ERK/MAPK signaling pathway.

### Nicotine Upregulates the VEGF/PEDF Ratio

**Figure 5:**
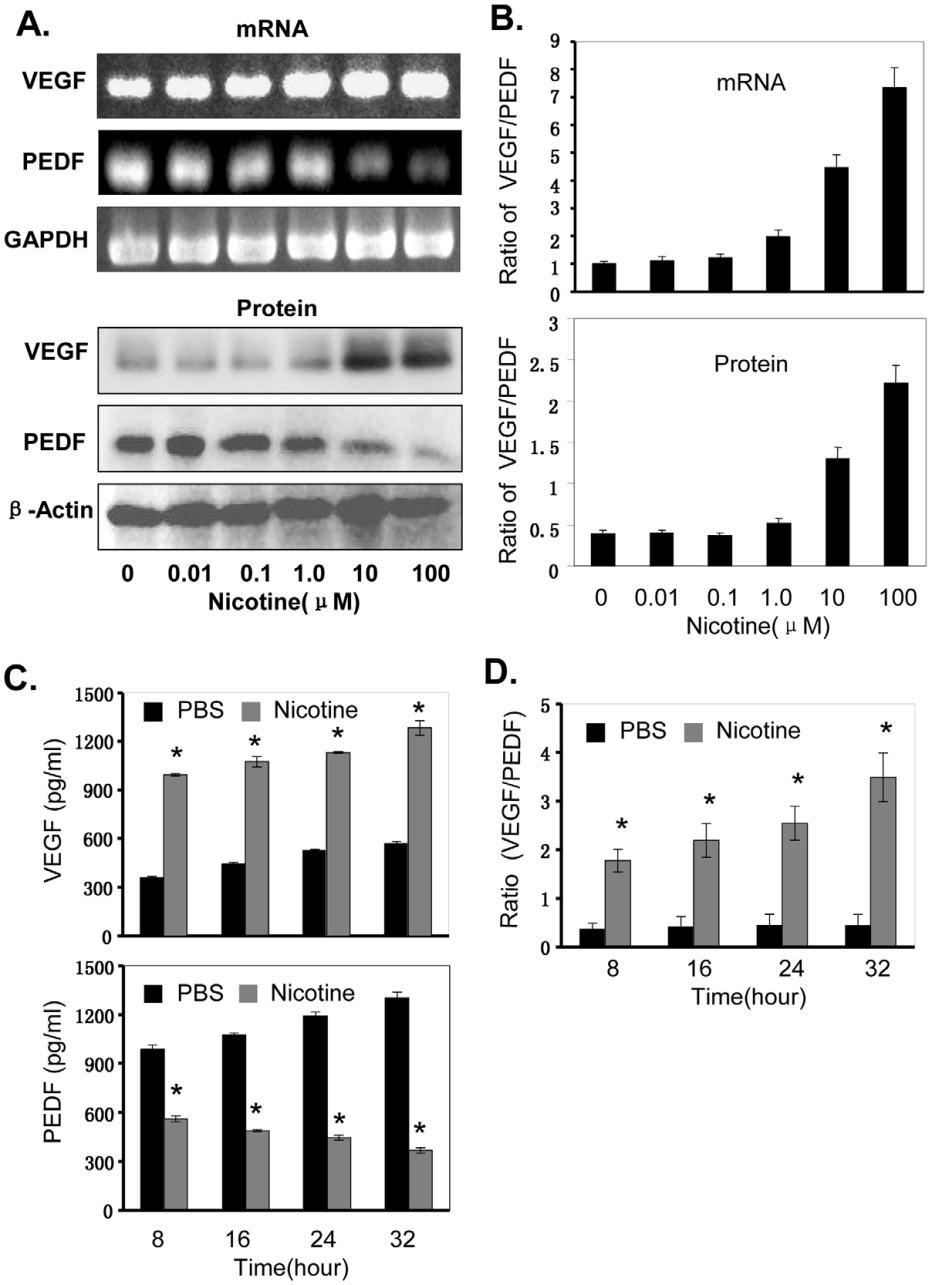
Nicotine increased the ratio of VEGF/PEDF in NPC cells. Human CNE1 cells were treated with nicotine at the doses of 0.01–100 µM for 24 hour **(A, B)** or at the concentration of 10 µM for 8–32 hours (**C, D**). The VEGF and PEGF expression at mRNA and protein levels **(A, B)** in cells and the release of VEGF and VEGF proteins in cell culture media **(C, D)** were determined by RT-PCR, Western blot and ELISA, respectively. The ratio of VEGF/PEDF was calculated. The data are presented as the mean ± SD of three separate experiments. *, *P*<0.05, significant differences between treatment groups and control groups.

HIF-1α has been shown to regulate transcription of VEGF and PEDF genes and the increased ratio of VEGF/PEDF is required for angiogenesis and tumor growth [19], [20]. We next determined the effect of nicotine on the expression of VEGF and PEDF at mRNA and protein levels in NPC cells by RT-PCR and Western blot, and on the release of VEGF and PEDF proteins in cell culture media by ELISA. As shown in Fig.5A and 5B, treatment of CNE1 cells with nicotine at the dose 0.01–100 µM considerably promoted the expression of VEGF mRNA and VEGF protein, but significantly decreased the expression levels of PEDF (Fig. 5A), leading to a significant increase of the VEGF/PEDF ratios in both mRNA and protein levels in a concentration-dependent manner (Fig. 5B). Similarly, treatment of NPC cells with nicotine (10 µM) for 8–32 hours also promoted the release of VEGF protein, but reduced the release of PEDF protein in cell culture media (Fig. 5C), resulting in a significant increase of the VEGF/PEDF ratio at protein level in cell culture media in a time-dependent manner (Fig. 5D). These results indicate that nicotine-induced cell proliferation might be also through the regulation of VEGF/PEDF ratio in NPC cells.

### Nicotine-mediated Promotion of VEGF/PEDF Ratio is α7AChR, ERK and HIF-1α-dependent

**Figure 6:**
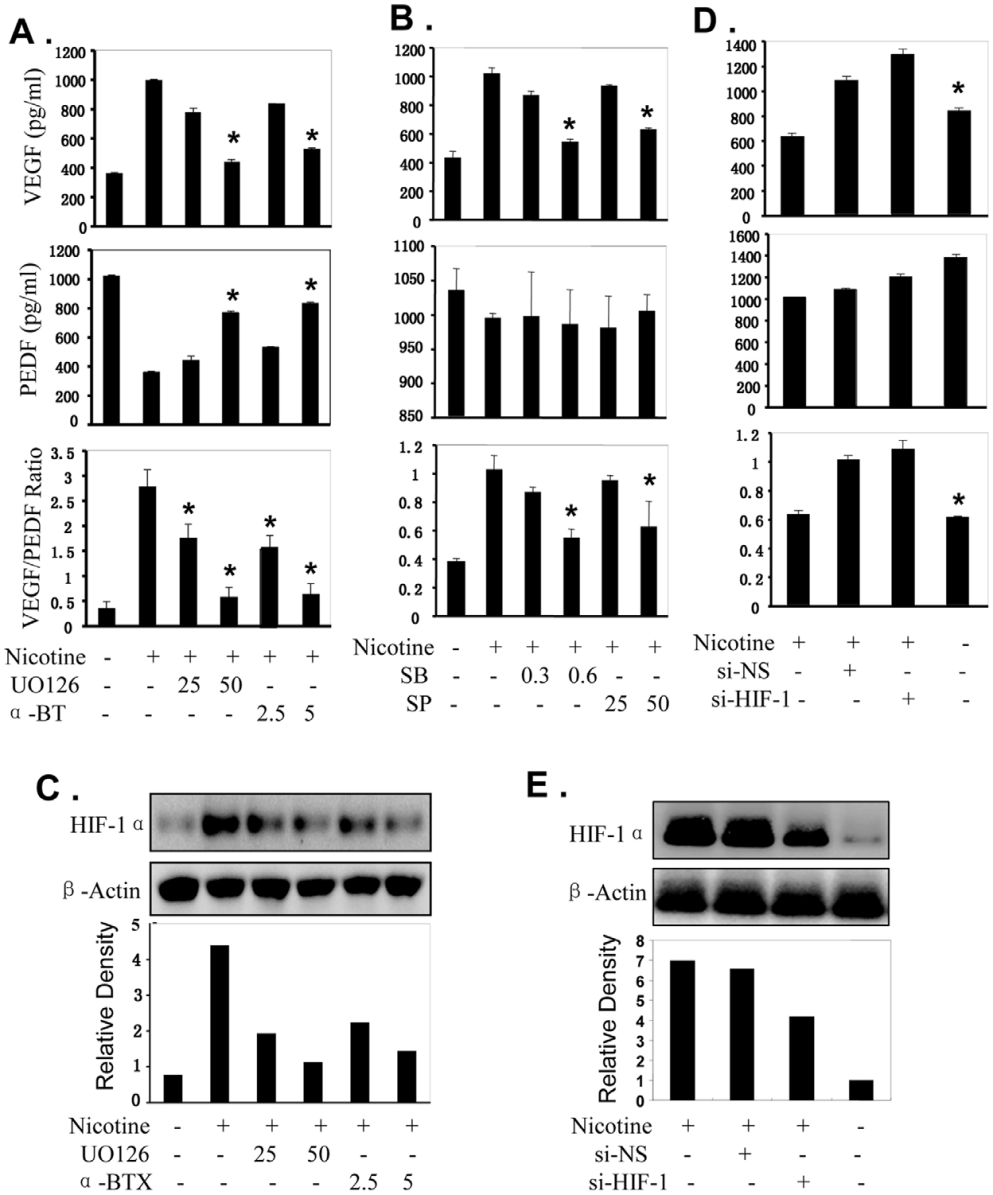
Nicotine-mediated increase of VEGF/PEDF ratio is α7AChR, ERK and HIF-1α-dependent. Human CNE1 cells were pretreated with α7AChR inhibitor α-BTX (2.5 and 5 µM) or ERK inhibitor UO126 (25 and 50 µM) (**A, C**), JNK inhibitor SB203580 (0.3 and 0.6 µM) or p38 inhibitor SP600125 (25 and 50 µM) (B)**,** HIF-1α-specific siRNA (si-HIF-1, 100 nM) or non-specific siRNA control (si-NS, 100 nM) (**D, E)** for 4 hours followed by nicotine (10 µM) treatment for 24 hours. The VEGF and PEDF protein levels in cell culture media (**A, B, D**) and the expression of HIF-1α proteins in NPC cells (**C, E)** were detected by ELISA and Western blot, respectively. The ratio of VEGF/PEDF was calculated (**A, B, D)**. The data are presented as the mean ± SD of three separate experiments. *, *P*<0.05, significant differences between treatment groups and control groups.

To determine whether the nicotine-mediated increase of VEGF/PEDF ratio is through regulating the α7AChR and ERK/MAPK signaling, we analyzed the effect of α7AChR or ERK-selective inhibitors on VEGF/PEDF ratio in nicotine-treated CNE1 cells. Pretreatment with a α7AChR or ERK-selective inhibitor dramatically attenuated the nicotine-mediated promotion of VEGF/PEDF ratio (Fig. 6A), whereas pretreatment with a JNK inhibitor (SB203580) or P38 inhibitor (SP600125) slightly affect the nicotine-mediated VEGF/PEDF ratio in NPC cells (Fig. 6B). Meantime, pretreatment of NPC cells with the α7AChR or ERK inhibitor effectively downregulated the expression of HIF-1α induced by nicotine (Fig. 6C). These results not only indicate the α7AChR and ERK signaling pathways play important roles in mediating nicotine’s effect on promoting VEGF/PEDF ratio in NPC cells, but also show that the 7AChR and ERK signaling mediates nicotine’s effect on promoting VEGF/PEDF ratio through upregulation of the HIF-1α signaling in NPC cells**.**

To further validate the role of nicotine in regulating VEGF/PEDF ratio through HIF-1α signaling, we blocked the expression of HIF-1α proteins by transfecting NPC cells with an HIF-1α-specific siRNA (si-HIF) and evaluated their effects on nicotine-mediated promotion of the VEGF/PEDF ratio. By comparison with the scrambled non-specific control siRNA (si-NS), transfection of CNE1 cells with the si-HIF dramatically inhibited the nicotine-mediated promotion of VEGF/PEDF ratio (Fig. 6D). Western blot analysis showed that HIF-1α siRNA markedly inhibited HIF-1α protein levels in CNE1 cells (Fig. 6E). These results indicate that nicotine increases VEGF/PEDF ratio through simultaneous modulation of the α7AChR, ERK and HIF-1α signaling.

## Discussion

In this study, we evaluated the response of human nasopharyngeal carcinoma (NPC) cells to nicotine treatment. Nicotine effectively promoted NPC cells proliferation in a time and dose-dependent manner. Our results showed that nicotine upregulated the expression of α7nAChR protein. The inhibition of α7nAChR by its specific siRNA or selective inhibitor significantly attenuated the nicotine-stimulated cell proliferation in NPC cells. We found that nicotine promoted the expression of HIF-1α protein, leading to an activation of downstream hypoxia-responsive genes associated with tumor cell proliferation. We also found that nicotine upregulated phophorylation of ERK1/2 protein and increased the ratio of VEGF/PEDF in NPC cells. Furthermore, we showed that the promotion of VEGF/PEDF ratio by nicotine in NPC cells is α7AChR, HIF-1α and ERK/MAPK-dependent. To our knowledge, this is the first report that demonstrated that nicotine exerted its effects in promoting cell proliferation through simultaneous upregulation of the a7ChR, HIF-1α, ERK and VEGF/PEDF signaling in human NPC cells.

α7AChR, the known receptor of nicotine, has been shown to mediate the effect of nicotine on angiogenesis, cell proliferation of endothelial cells as well as lung carcinoma cell lines *in vitro* and *in vivo* [28]. By binding to the nAChRs, nicotine exerts its biological effects through activation of a number of signaling pathways, including the influx of Ca^2+^ and activation of calmodulin, PKC [29], PI3K/Akt [30], and Raf-1/MAPK/ERK1/2 [31]. In our study, we also demonstrated the promotion effects of nicotine on α7AChR protein expression in NPC cells, and demonstrated that blocking the activation of α7AChR by siRNA or a specific inhibitor effectively attenuated the nicotine-mediated promotion of NPC cell proliferation. These results suggest that nicotine promotes NPC cell proliferation partially through the mechanisms by which nicotine modulates the α7AChR signaling.

HIF-1α pathway exists as a critical step in carcinogenesis [32], [33], which was implied by a growing body of evidence due to its linkage to several oncogenic and tumor suppressor gene pathways in cancer. As a transcription factor, HIF-1α heterodimerizes with the constitutively expressed HIF-1ß subunit, and they activate the expression of a number of genes, including VEGF, to take part in tumor angiogenesis and tumor cell proliferation and invasion. Our present study also showed that HIF-1α played a crucial role in regulating NPC cells growth mediated by nicotine. It is possible that nicotine promotes HIF-1α protein accumulation in NPC cells, and then HIF-1α induces the expression of the related genes which are involved in tumor cell proliferation. Thus, our results show that HIF-1α contributes, at least in part, to nicotine-induced NPC cells proliferation.

The MAPK signaling pathway plays a key role in the regulation of gene expression, cellular growth, and survival. Abnormal MAPK signaling may lead to increased/uncontrolled cell proliferation, resistance to apoptosis and to chemotherapy, radiotherapy, and targeting therapies in tumors [34], [35]. The relationship of MAPK to cancer is an intense research area nowadays. The activation of HIF-1 protein could be facilitated by MAPK signaling through p300/CBP. As the upstream signaling molecules of HIF protein, MAPK might also play its role in mediating the effect of nicotine on the promotion of NPC cells growth. Our research demonstrated the important role of MAPK signaling pathway in nicotine-mediated promotion of cell growth. By comparison with JNK and p38 MAPK signaling pathways, ERK/MAPK signaling pathway plays more important role in nicotine-mediated promotion of cell proliferation. Nicotine might function through activating ERK/MAPK signaling pathway, promoting the phosphorylation of ERK protein, and upregulating HIF-1α signaling to promote tumor cell proliferation.

VEGF has been recognized as one of the principal initiators of tumor angiogenesis. Its expression is regulated by a body of external factors, of which hypoxia is the best characterized mediator. By binding to the hypoxia-responsive elements on VEGF promoter, HIF1 leads to the transcriptional activation of the VEGF gene [36]. The potent angiogenic inhibitor pigment epithelium-derived factor (PEDF) counterbalances the effect of VEGF [37]. Although previous studies have reported that nicotine stimulates VEGF expression in some kinds of cancer cells, including gastric tumor [38], [39] and cervical cancer cell lines [40], the underlying mechanisms remain poorly known. Consistent with previous studies, our study also showed that nicotine stimulated VEGF production, lowered PEDF expression, and significantly increased VEGF/PEDF ratio in NPC cells**.** The increase of VEGF/PEDF ratio can be inhibited by the selective inhibitors of α7AChR and ERK, indicating that nicotine-mediated increase of VEGF/PEDF ratio is α7AChR and ERK-dependent. Furthermore, we found that the activity inhibition of α7AChR or ERK also attenuated the increased expression of HIF-1α mediated by nicotine, suggesting that the role of α7AChR and ERK in regulating nicotine-mediated promotion of NPC cells proliferation might be realized through activating HIF-1α. The inhibition of VEGF/PEDF ratio in NPC cells through disrupting HIF-1α expression using siRNA strategy not only demonstrated nicotine-mediated increase of VEGF/PEDF ratio is HIF-1α-dependent, but also showed HIF-1α’s central role in regulating the VEGF/PEDF ratio stimulated by nicotine in human NPC cells. Further studies are needed to elucidate the mechanism by which nicotine simultaneously modulating multiple signaling pathways involving in cell proliferation.

**Figure 7:**
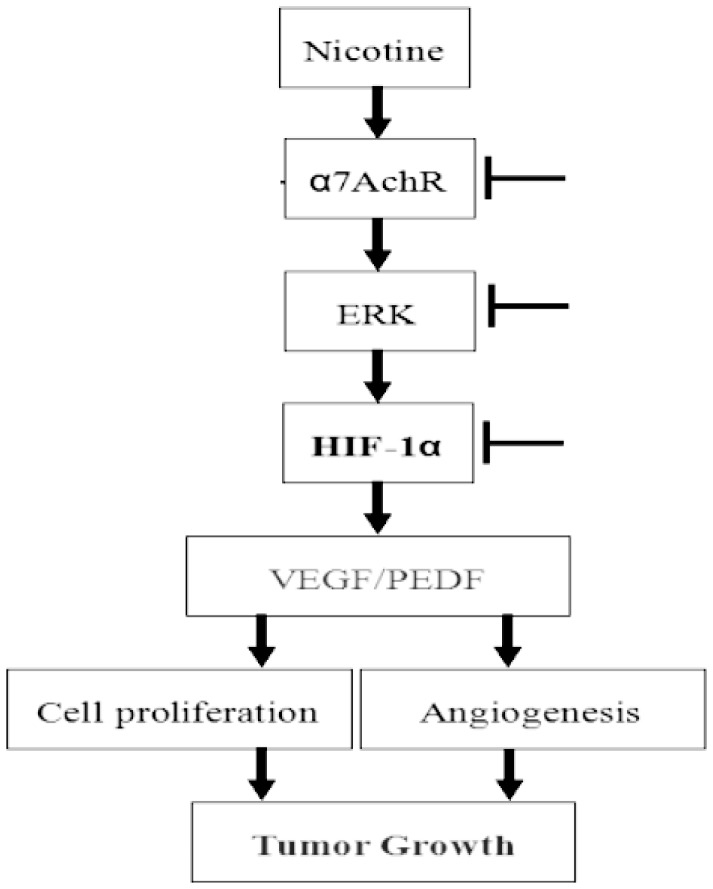
The proposed mechanisms by which nicotine promotes NPC cell proliferation. Nicotine promotes NPC cell proliferation by simultaneously promoting the α7AChR, ERK, HIF-1α and VEGF/PEDF signaling. Nicotine interacts with α7AChR on the surface of NPC cells, activates ERK/MAPK signaling pathway, upregulates HIF-1α expression, elevates VEGF/PEDF ratio, thereby promotes NPC cells proliferation.

In summary, we demonstrated that nicotine promoted NPC cell proliferation by simultaneously upregulating the α7AChR, HIF-1α, ERK/MAPK and VEGF/PEDF signaling. These findings provide new insights into the possible molecular mechanisms of nicotine-mediated promotion of NPC cell proliferation (Fig. 7). Nicotine interacts with α7AChR on the surface of NPC cells, activates ERK/MAPK signaling pathway, upregulates HIF-1α signaling, elevates VEGF/PEDF ratio, thereby promotes NPC cells proliferation (Fig. 7). HIF-1α signaling pathway might exert a central effect in the nicotine-mediated promotion of cell proliferation, suggesting that HIF-1α might be a promising target for the treatment of human NPC. Our study also provides some clues for the development of anticancer therapy in tobacco-associated human diseases such as nasopharyngeal carcinomas.

## Materials and Methods

### Cell Lines and Cell Culture

Human NPC cell lines CNE1 (human nasopharyngeal high differentiated squamous epithelium carcinoma cell), CNE2 (human nasopharyngeal low differentiated squamous epithelium carcinomas cell) and its clones (S18 and S26) were cultured in RPMI1640 medium (Invitrogen, Carlsbad, CA), supplemented with 10% fetal bovine serum (FBS) (HyClone, Logan, UT), 5% glutamine, 100 U/ml penicillin and 100 µg/ml streptomycin (Invitrogen, Carlsbad, CA). In all experiments, 60–70% of confluent cells were washed and incubated in serum-free medium for 24 hours prior to treatment with nicotine, α-BTX, U0126 (Sigma, St. Louis, MO), SB203580 or SP600125 (Beyontime Institute Technology, Shanghai, China) for the indicated time.

### Cell Viability Assay

Cell viability was determined by the MTT assay (Roche Diagnosis, Indianapolis, IN) as previously described [41]–[43]. Briefly, cells plated in 96-well plates (2000 cells/well) were treated with nicotine at the indicated doses. At 72 hours after treatment, cell viability was determined.

### Western Blot Analysis

Cell lysate proteins were separated by electrophoresis in a 10% sodium dodecyl suplhaste-polyacrylamide gradient minigel (SDS-PAGE) (Bio-Rad, Hercules, CA) and electrophoretically transferred to a nitrocellulose membrane (Amersham Pharmacia Biotech, Piscataway, NJ). Western blots were probed with antibodies against β-Actin, α7AChR, HIF-1α (Santa Cruz Biotechnology, Santa Cruz, CA), pTyr202/Y204-ERK1/2, ERK1/2, pThr180/Tyr182-p38 MAPK, p38 MAPK, pThr183/Tyr185-SAPK/JNK, SAPK/JNK (Cell Signaling, Beverly, MA). The protein bands were detected by enhanced chemiluminescence (Amersham Pharmacia, Piscataway, NJ).

### Reverse Transcription-polymerase Chain Reaction (RT-PCR)

Total RNA was extracted with Tri-Zol reagent (Life Technologies, Glasgow, UK) according to the manufacturer's instructions. cDNA was extracted and used for amplification of VEGF and PEDF genes. The amplified products were visualized on 1% agarose gels.

### Determination of VEGF and PEDF Production in Cell Culture Media

The cells were seeded in 96-well plates and treated with nicotine at the indicated concentrations. VEGF and PEDF levels in cell culture media were quantified using a VEGF Immunoassay Kit (968962) (R & D Systems, Minneapolis, MN) and a Chemikine PEDF ELISA Kit (CYT420) (Chemikine, Billerica, MA) according to the manufacturer's protocols.

### siRNAs

The α7AChR and HIF-1α siRNAs were purchased from Santa Cruz Biotechnology (Santa Cruz, CO). Cells were transfected with siRNA duplexes (100 nM) using the Oligofectamine reagent (Invitrogen, Carlsbad, CA).

### Densitometric Analysis

Molecular Image system (Kodak, Rochester, NY) was used to determine the density of protein or mRNA bands detected by Western blots and RT-PCR. The data are expressed as an arbitrary unit.

### Statistical Analysis

Analysis of variance and Student’s *t* test were used to compare the values of the test and control samples. *P<0.05* was considered to a statistically significant difference. SPSS6.0 software was used for all statistical analysis. The significance was evaluated by the paired *t* test. All the experiments were done three times, and mean values and standard deviation were calculated.
